# Bacterial cellulose nanofibers decorated with graphene/Cu-Mg MOF for sorption of zirconium, yttrium, and strontium ions from multicomponent system

**DOI:** 10.1186/s13065-026-01774-5

**Published:** 2026-04-09

**Authors:** E. M. Abu Elgoud, Aya M. Matloob, Deyaa Abol-Fotouh, H. F. Aly, Ola E. A. Al-Hagar

**Affiliations:** 1 Nuclear Fuel Chemistry Department, Hot Laboratories Center, Egyptian Atomic Energy Authority, Cairo, 13759 Egypt; 2 Refining Department, Egyptian Petroleum Research Institute (EPRI), Naser City, Cairo, 11727 Egypt; 3 Advanced Technology and New Materials Research Institute (ATNMRI), City of Scientific Research and Technological Applications (SRTA-City), New Borg El-Arab City, Alexandria, 21934 Egypt; 4 Plant Research Department, Nuclear Research Center, Egyptian Atomic Energy Authority, Cairo, 13759 Egypt

**Keywords:** Sustainable polymer, Nanocomposite, In situ synthesis, MOF, Zirconium, Yttrium, Strontium, Sorption, Multi-component system

## Abstract

Bacterial cellulose (BC) composites attained elevated application areas owing to the distinguished physical properties offered by this sustainable polymer. The uncontrolled release of isotopes into aquatic systems through diverse industrial effluents constitutes a pressing environmental concern, necessitating the development of advanced functional materials engineered with superior properties and sustainable nature to efficiently capture and immobilize these isotopes, thereby mitigating ecological risks and safeguarding public health. In this study, one-pot in situ route was utilized to synthesize a nanocomposite composed of bacterial cellulose/graphene/bimetallic copper-magnesium metal-organic framework (BC/Gr/Cu-Mg MOF). The synthesized nanocomposite was characterized for its structural properties by the Fourier Transform Infrared Spectroscopy (FTIR), X-ray diffraction (XRD), Scanning Electron Microscope (SEM), Transmission Electron Microscope (TEM), Energy Dispersive Spectroscopy (EDS), and Brunauer-Emmett-Teller Method (BET) for determining surface area. The results proved the synthesis of the Gr/Cu-Mg MOF successfully around the BC nanofibers. The BC/Gr/Cu-Mg MOF was initially inspected for its sorption ability to Zr(IV), Y(III), and Sr(II) utilizing a multi-component system. The results indicated that the nanocomposite has the potential for the selective sorption and separation of these metal ions. Batch adsorption experiments demonstrated empirical adsorption capacities of 178.05, 50.67, and 7.275 mg/g for Zr(IV), Y(III), and Sr(II), respectively. The equilibrium data were well described by the Langmuir isotherm model. The thermodynamic analysis revealed that the sorption of Zr^4+^, Y^3+^ and Sr^2+^ by the BC/Gr/Cu-Mg MOF is endothermic and occurs spontaneously. These outcomes manifested the feasibility of both the one-pot synthesis strategy, and the sorption capability of the examined green nanocomposite as well.

**Graphical Abstract:**

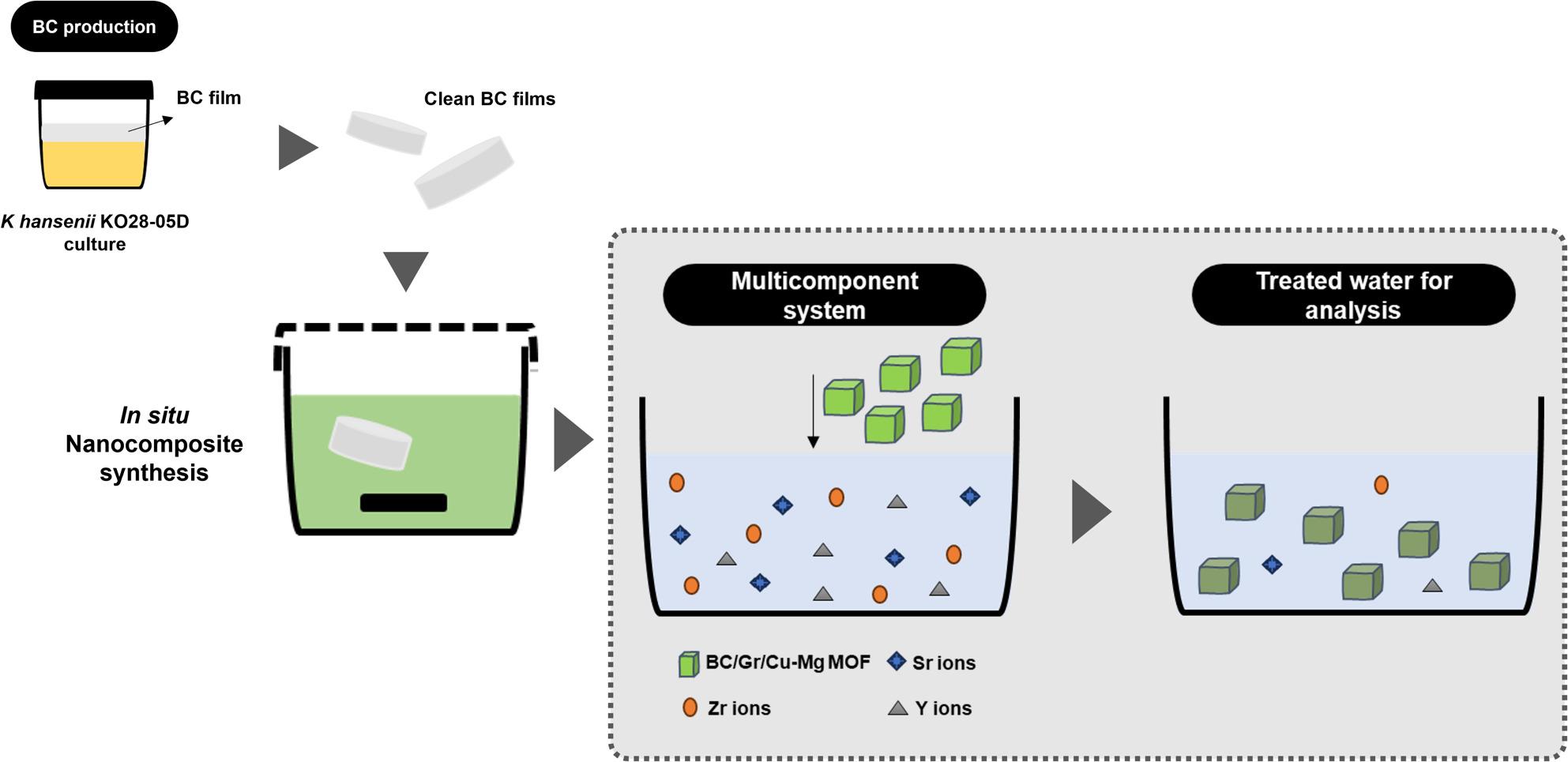

**Supplementary Information:**

The online version contains supplementary material available at 10.1186/s13065-026-01774-5.

## Introduction

Zirconium and yttrium are critical elements for nuclear applications, and their demand has increased significantly [1]. The most significant yttrium isotopes are ^90^Y and ^91^Y, related to the fission products ^90^Sr and ^91^Sr, generated from β-decay. ^90^Y is a pure beta emitter, and it has a short half-life of approximately 64.4 h and decays into a stable daughter isotope, ^90^Zr [2]. In radionuclide therapy, ^90^Y is being used extensively to treat numerous types of cancer, especially when irradiating solid tumors. Zirconium-89 is a radiotracer that is widely used in several fields of interest.

Among the medical uses are pharmacokinetics, tumor cell treatment, immunoimaging, cell imaging, and antibody imaging [3, 4]. In the nuclear field, zirconium is particularly valuable due to its unique combination of low absorption cross-section for neutrons with good mechanical qualities, high corrosion resistance, and, most importantly, remarkable resistance to water under pressure and to steam at higher temperatures. The separation of zirconium from yttrium is essential due to the importance of purifying zirconium from yttrium for use in the applications mentioned above.

Many chemical separation procedures have been utilized, such as solvent extraction, extraction chromatography, ion exchange, adsorption, and precipitation. Various adsorbents have been used to adsorb and separate zirconium from yttrium. For example, Amberlite XAD–4 resin impregnated with Cyanex 572 [5], and mesoporous titanium dioxide and sodium modified titanium dioxide [6].Mansel and Franke optimized the liquid-liquid extraction process of zirconium from lanthanides using a radiotracer technique [1].

The radionuclide, ^89^Zr, is produced by proton irradiation of a metallic yttrium target and purified using UTEVA resin [7]. Ivanov et al. explored the use of high energy α-particles to produce ^89^Zr, by irradiating a thick ^nat^SrO target with charged particles, zirconium-88,89 was produced [8].

Metal–organic frameworks (MOFs) are a type of inorganic-organic hybrid materials with porous, reticular and crystalline structures [9, 10]. The unique arrangement of metal and ligands determine their diversified structural features. A lot of metals and organic ligands could be used to engineer MOFs with distinct properties for a wide range of applications including gas separation [11], gas storage/adsorption [12], catalysis [13], drug delivery [14], bioimaging [15], and phototherapy [16].

Interestingly, elevated surface area and abundance of accessible sites recommending MOFs as highly efficient adsorbing materials [17], especially copper-based MOFs which exert distinguished physico-chemical characteristics among MOFs, grabbing escalated attention for removing different pollutants from water.

The incorporation of a bimetallic system, specifically copper and magnesium, in the synthesis of metal-organic frameworks (MOFs) offers distinct advantages over monometallic counterparts. The synergistic interplay between Cu^2+^ and Mg^2+^ ions enhances both the adsorption selectivity and overall sorption capacity. Copper ions contribute to stronger coordination interactions with target species, while magnesium ions improve structural stability and modulate surface characteristics. The presence of dual metal centers introduces heterogeneous active sites, thereby facilitating multivalent binding interactions particularly beneficial in systems involving Zr(IV), Y(III), and Sr(II) [18]. Moreover, the bimetallic configuration influences the pore architecture, resulting in increased surface area and optimized pore size distribution, as evidenced by Brunauer–Emmett–Teller (BET) analysis. Collectively, Cu-Mg MOFs exhibit superior structural integrity, tunable functionality, and enhanced adsorption performance relative to their single-metal analogs [19].

Bacterial cellulose (BC) has garnered growing global attention due to its exceptional physical and chemical characteristics. These include environmentally friendly processing routes, low production costs, outstanding mechanical strength, hydrophilicity, and minimal carbon footprint, positioning BC as a sustainable and high performing biomaterial [20, 21]. Unlike plant-derived cellulose, BC is synthesized in a highly pure form, devoid of lignin, pectin, and hemicelluloses. Its ultrafine nanofibrous structure offers superior crystallinity, enhanced liquid absorption capacity, a higher degree of polymerization, increased specific surface area, and elevated mechanical properties—making it a more advantageous alternative to plant cellulose across various industrial and biomedical applications [22]. In a recent advancement, our research team successfully harnessed the mutagenic potential of low doses gamma irradiation to significantly boost BC production. By applying controlled doses to the locally isolated strain *Komagataeibacter hansenii* KO28-05D, more than fourfold rise in BC yield was achieved under identical cultivation conditions, demonstrating the efficacy of radiation-induced strain enhancement [23].

Beyond its structural merits, BC has also proven effective as a functional matrix in environmental remediation. BC-based nanocomposites have been extensively investigated for their ability to adsorb a wide range of water pollutants, including organic dyes, pharmaceutical residues, and heavy metals [24, 25]. For example, Maged et al. developed a one-step biosynthesis approach to fabricate a BC/kaolinite nanocomposite, which exhibited notable adsorption capacities for basic blue 9 dye (Bb9) and norfloxacin (NFX) antibiotic reaching 127.6 mg/g and 101.6 mg/g, respectively [26]. Furthermore, a BC/Cu-Fe-CN composite demonstrated a high sorption capacity of 160.51 mg/g for cesium ions (Cs^+^) in batch experiments, following optimization of the adsorption conditions [27]. Elias et al. have demonstrated the effectiveness of bacterial cellulose (BC) in enhancing chitosan-based adsorbents for wastewater treatment. BC-reinforced CS/PVA beads and chitosan/polyethyleneimine composites showed high adsorption capacities for antibiotics such as vancomycin, azithromycin [28], tetracycline, and metronidazole [29], as well as for dyes like Congo red [30], with rapid uptake and excellent reusability.

Enforcing BC with different compounds such as carbon nanotubes [31], graphene oxide (GO) [32], and MOFs [33] was widely studied for several implementations. Graphene (Gr) exerts high reactive surface area with a varied range of functional groups, high thermal stability, and chemical resilience, enabling it to maintain its impressive reactivity even in the extreme conditions [25]. Several reports have studied compositing BC, graphene and/or MOFs through either *ex-situ* or in situ strategies. The *ex-situ* method includes incorporating the already synthesized graphene and /or MOFs fractions inside the already synthesized BC matrix [32, 33], while the in situ route includes addition of graphene/MOFs fractions to the BC synthesis pot. For instance, Dhar and his group investigated the formation of BC/Gr through a single step in situ by introducing GO into the BC-producing bacterial culture to be anchored throughout the formed nanofibers and reduced to Gr simultaneously [34].

Moreover, the in situ strategy can include introducing the already-synthesized BC material during the graphene and/or MOF synthesis phase to result in the composite formation. An evident example is what was reported by Ashour et al.., which synthesized MIL-100(Fe)@BC nanocomposite through one-pot route included mixing the BC material with the reactants during the MOF- synthesis session [35].

The principal objective of this study is to synthesize chemically stable and reactive BC/Gr/Cu-Mg MOF nanocomposite through in situ synthesis route and evaluating its capability for the first time, in the best of our knowledge, for the sorption and separation of Zr(IV), Y(III) and Sr(II) from multi-component system. The proposed synthesis strategy was designed to yield a multifunctional nanocomposite that integrates the structural advantages of bacterial cellulose (BC) with the high surface reactivity of graphene (Gr) and metal–organic frameworks (MOFs). The resulting BC/Gr/Cu-Mg MOF composite was anticipated to inherit the mechanical robustness, crystallinity, hydrophilicity, and porosity characteristic of BC, while also benefiting from the elevated chemical reactivity and extensive surface area provided by the incorporated Gr and MOF components.

To assess its sorption performance, a comprehensive investigation was conducted examining the effects of key operational parameters including shaking time, solution pH, initial metal ion concentration, adsorbent dosage, and temperature on the adsorption behavior. The evaluation was supported by advanced characterization techniques such as energy-dispersive X-ray (EDX) mapping, scanning electron microscopy (SEM), and Fourier-transform infrared spectroscopy (FTIR), performed both before and after the adsorption process. These analyses provided critical insights into the composite’s structural integrity and functional group interactions, confirming its efficacy in adsorbing zirconium (Zr), yttrium (Y), and strontium (Sr) ions from multicomponent aqueous systems.

## Materials and methods

### Materials and chemicals

The chemicals used in this work were of analytical grade and applied without further treatment.

Cu(NO_3_)_2_⋅3H_2_O, MgNO_3_, graphite powder, 1,3,5-Benzene tricarboxylic acid (BTC, 98%), SrCl_2_·6H_2_O, and Y_2_O_3_ (Sigma-Aldrich, ≥ 99%), HNO_3_ (Sigma-Aldrich, 65%), HCl (30%; El Salam Chemical Industries, Egypt, analytical grade), Zirconyl chloride octahydrate (ZrOCl_2_·8H_2_O, ≥ 98%) and ethanol (96%) (Alfa Aesar), D-Glucose (Fisher^®^ Chemicals, USA, ≥ 99%), citric acid (Fisher^®^ Chemicals, USA, ≥ 99%), and peptone, yeast extract, and agar (Grisp^®^ supplies, Portugal, analytical grade) were all of analytical grade and used without further purification.

### Methodology

#### Bacterial cellulose (BC) production

BC films were produced using the strain *Komagataeibacter hansenii* KO28-05D, originally isolated from a local Egyptian environment and identified at the Microbiology Lab, Nuclear Research Center, EAEA. In a prior study, the strain underwent dual low-dose gamma irradiation (0.5 kGy) using a ^60^Co source (MC20 irradiator, Russia) at a dose rate of 0.77 kGy/h to induce genetic mutation and enhance BC yield [23, 36]. The resulting mutant, KO28-05D, was selected as the optimized BC-producing strain.

BC films were synthesized by propagating the strain *K. hansenii* KO28-05D on the production medium classically designated as Hestrin-Schramm (HS) and constitutes (% w/v): D-glucose (2), yeast extract (0.5), peptone (0.5), yeast extract (0.5), Na_2_HPO_4_ (0.27), and citric acid (0.115) [37]. A sterile beaker of 100 mL capacity was filled with 18.5 mL of autoclaved HS medium and supplied with 1.5 mL of the seven-days-age strain inocula. The mixture was blended gently before the beaker was sealed and incubated at 30 °C for 7 days in darkness. Incubation was ended by collecting the newly formed topical BC films and underwent washing phases, which includes applying hot water and hot 0.1 N NaOH for 10 min (twice for each), before the extensive rinsing by dH_2_O was implemented to restore the neutral pH to the clean BC films. The pristine BC hydrogel films then were stored in dH_2_O at 4 ℃ until further demand.

#### In situ synthesis of BC/Gr/Cu-Mg MOF nanocomposite

Graphene oxide (GO) was synthesized *via* Hummers’ method with certain modification [10]. Specifically, 3 g of graphite was dispersed in a mixture of concentrated sulfuric acid (98%, 70 mL) and phosphoric acid (85%, 6.7 mL) under continuous stirring in an ice bath for 30 min. Subsequently, potassium permanganate, in proportion to the amount of graphite used, was gradually introduced under vigorous agitation. The suspension was then maintained under stirring at ambient temperature for 24 h, during which a dark green dispersion evolved into a highly viscous mixture, indicating the oxidation of graphite and the concurrent formation of manganese dioxide. To terminate the reaction, 500 mL of deionized water was added at 95 °C under stirring for 15 min, followed by the addition of 15 mL hydrogen peroxide until a yellow coloration appeared. The mixture was allowed to stand for at least 12 h, after which the clear supernatant was decanted. The residual precipitate was thoroughly washed with hot deionized water. Finally, the metallic grey graphene oxide (GO) precipitate was collected and dried at room temperature.

Two sheets of BC were treated with 1 M of NaOH and stirred for 90 min at 70 °C, before the sheets were immersed in a liquid mix included: 1 mmol Cu(NO_3_)_2_⋅6H_2_O solution; 1 mmol L^− 1^ of MgNO_3_⋅6H_2_O solution; and GO (0.2 gm was ultrasonicated in ethanol: water 2:1 for 30 min). Additionally, 1 mmol L^− 1^ of 1,3,5-Benzene tricarboxylic acid (BTC) solution was added under sonication. After 30 min of sonication, the resulted solution was kept under stirring for 12 h at 100 °C, then the sheets were collected, washed excessively with ethanol, before they stored in ethanol until demand.

#### Characterization of the synthesized BC/Gr/Cu MOF films

The chemical functionalities of the samples were analyzed using Fourier Transform Infrared Spectroscopy (ALPHA II Compact FTIR, Bruker, Germany) over the 4000–400 cm⁻¹ range, with a resolution of 2 cm⁻¹ and 16 scans per measurement. Crystallographic structures of BC, Gr/Cu MOF, and BC/Gr/Cu MOF were examined *via* X-ray diffraction (labX XRD-6100, Shimadzu, Japan) using CuKα radiation (λ = 1.54 Å) at 40 kV and 30 mA, scanned across a 2θ range of 5°–80° at 0.5°/min.

Surface morphology was investigated using Scanning Electron Microscope (SEM) (Zeiss™ Evo 15, Germany) at 25 kV after sputter-coating samples with a 1 nm gold layer for 2 min. Transmission Electron Microscope (TEM) (Jeol 2100 Plus) was employed to visualize the nano-architecture of Gr/Cu MOF and BC/Gr/Cu MOF.

Textural properties, including Brunauer-Emmett-Teller Method (BET) surface area, pore size, and pore volume, were measured using a BELSORP-miniX analyzer after degassing the samples at 150 °C under vacuum. Mechanical properties of BC and BC/Gr/Cu-Mg MOF films were evaluated *via* uniaxial tensile testing (Ulm, Germany). Rectangular specimens (40 × 10 mm) were mounted on paper frames and tested at a strain rate of 10 mm/min with a 20 mm gauge length and 50 N load cell. Results were averaged over six replicates and reported as mean ± standard deviation.

#### Batch adsorption studies

A 1000 mg/L stock solution was prepared by dissolving appropriate amounts of each metal ion in double-distilled water. Batch sorption experiments were conducted to evaluate the influence of key parameters including shaking time, pH, adsorbent dosage, initial metal concentration, and temperature on the adsorption of Zr(IV), Y(III), and Sr(II) ions. Optimal conditions were identified through systematic variation. Strontium concentrations before (C_o_) and after adsorption (C_e_) were quantified using atomic absorption spectrophotometry, while Zr(IV) and Y(III) levels were determined *via* the Arsenazo III spectrophotometric method [38]. The sorption percentage, S%, of the Zr(IV), Y(III) and Sr(II) at equilibrium was calculated as given in Eq. 1. The batch procedure was implemented in compliance with the specifications listed in Table 1.

1 $$S\%=\left(\frac{{C}_{o}-{C}_{e}}{{C}_{o}}\right)*100$$

where *C*_*o*_ and *C*_*e*_ are the initial and equilibrium concentrations (mg/L) of metals ions in solution, respectively.

**Table 1:** Experimental conditions for Zr^4+^, Y^3+^, and Sr^2+^ sorption onto BC/Gr/Cu-Mg MOF

Batch sorption study					
Effect of different parameters	pH	Shaking time, min	[M], mg/L	adsorbent dosage, mg	Temperature, K
Effect of solution pH	1,2,3,4, and 5	60.0	100.0	20	298
Effect of shaking time	3.0	1,5,10,15,30,60, and 120	100.0	20	298
Effect of metal ions concentration	3.0	15.0	100,200,400,600,800, and 1000	20	298
Effect of adsorbent dosage	3.0	15.0	100.0	5, 10, 15,20, and 25	298
Effect of solution Temperature	3.0	15.0	100.0	10	298,308,318,328, and 338

#### Mathematical models

Table 2 describes the kinetics and isotherm models used in this investigation. Both the pseudo-first-order, pseudo-second-order and Elovich equations were used to analyze the sorption kinetics. The experimental results were validated by using the Langmuir, Freundlich, Temkin, and Halsey isotherm models. This comprehensive approach ensures a thorough understanding of the adsorption process.

**Table 2:** Linear forms of adsorption models

Isotherm	Linear form
Langmuir	$\frac{{C}_{e}}{{q}_{e}}=\left(\frac{1}{{Q}_{o}b}\right)+\left(\frac{1}{{Q}_{o}}\right){C}_{e}$
Freundlich	${log}{q}_{e}={log}{K}_{f}+\frac{1}{n}{log}{C}_{e}$
Temkin	${q}_{e}=Bln{K}_{t}+Bln{C}_{e}$
Halsey	${ln}{q}_{e}=\frac{1}{{n}_{H}}{ln}{K}_{H}-\frac{1}{{n}_{H}}ln{C}_{e}$
Kinetic model	
Pseudo-first -order	$\mathrm{log}\left({q}_{e}-{q}_{t}\right)=log{q}_{e}-\frac{{K}_{1}}{2.303}t$
Pseudo-second-order	$\frac{\mathrm{t}}{{q}_{t}}=\frac{1}{{K}_{2}{q}_{e}^{2}}+\frac{t}{{q}_{e}}$
Elovich kinetic	${q}_{t}=\frac{1}{{\upalpha}}{ln}\left({\upalpha}{\upbeta}\right)+\frac{1}{{\upalpha}}ln{\mathrm{t}}$

## Results and discussion

### In situ synthesis of Gr/Cu-Mg MOF nanocomposite

**Fig. 1:**
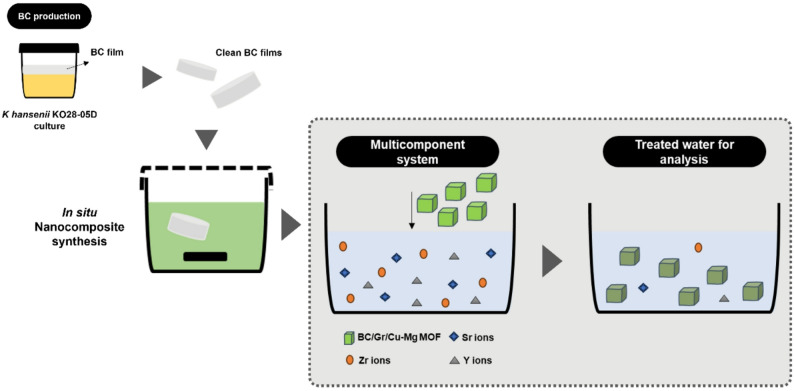
Schematic diagram pinpoints the phases of production of the BC/Gr/Cu-Mg MOF nanocomposite and examining its capability for adsorbing Zr(IV), Y(III) and Sr(II) ions through multicomponent system

In the present study, placing of the BC film inside the autoclave which harbors the Gr/Cu-Mg MOF synthesis reaction seems to induce the proceeding of the MOF formation around the BC nanofibers. The 3D nano porous nature of the BC hydrogel supposed to permit the reactants to flow through the entangled nanofibers, where the BC fibrous network represents a platform for the formation and localization of the MOF particles scattered throughout the whole fibers. Upon harvesting the BC film from the autoclave after terminating the reaction, the color turned dark grey as a sign of the Gr/Cu-Mg MOF formation. Figures 1 elucidates the phases of production and adsorption examination.

### Characterization of the synthesized BC/Gr/Cu-Mg MOF nanocomposite

**Fig. 2:**
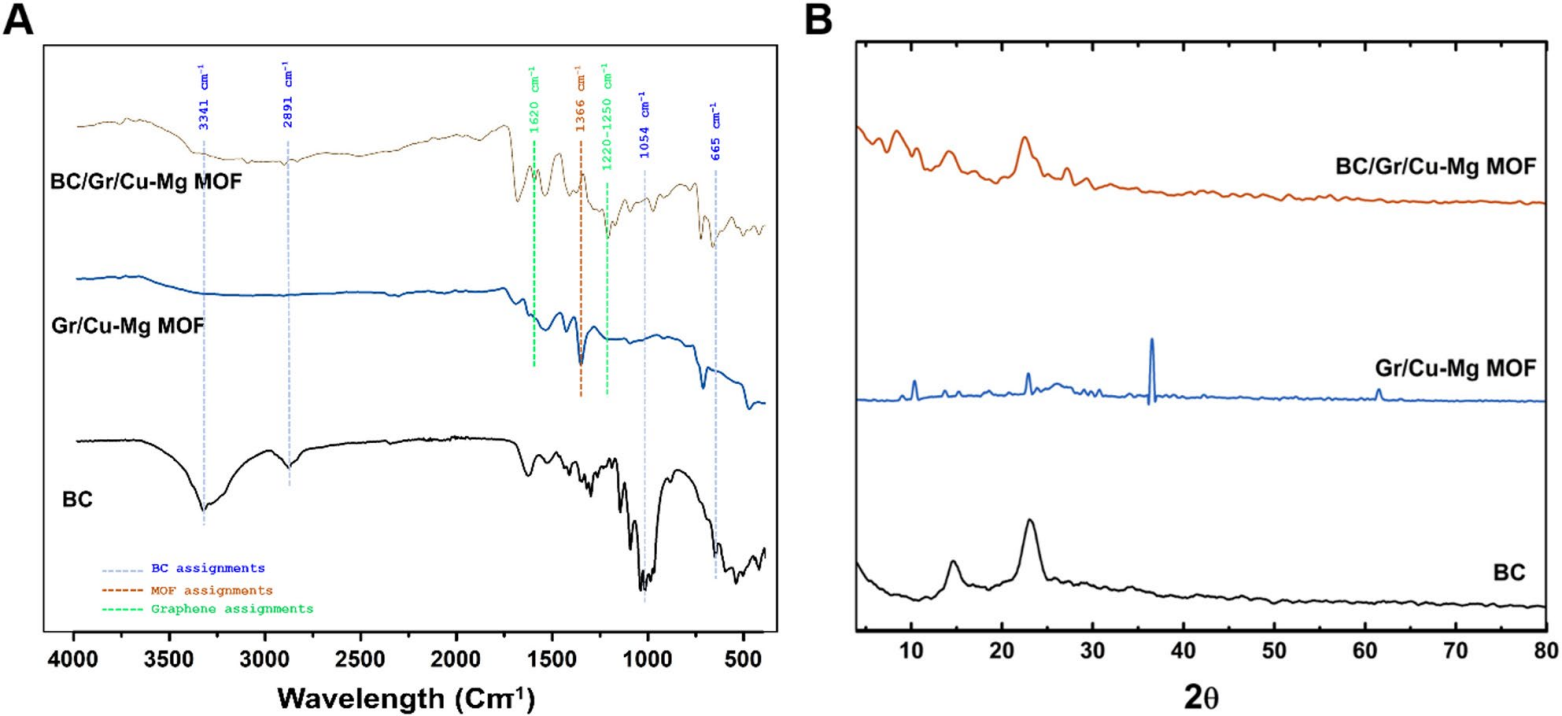
FTIR (**A**) and XRD diffraction (**B**) of the BC, Gr/Cu-Mg MOF, and BC/Gr/Cu-Mg MOF samples

The FTIR testing for the BC, Gr/Cu-Mg MOF, and BC/Gr/Cu-Mg MOF samples elaborated the successful generation of the Gr/Cu-Mg MOF throughout the whole BC film. In Figure 2A, marginal decrease in intensity of bands of cellulose and presence of MOF-related bands suggest that the.

Gr/Cu-Mg MOF nanoparticles were well dispersed and anchored within BC nanofiber network.

A general look on the IR patterns of the BC and BC/Gr/Cu-Mg MOF pinpoints that the nanocomposite sample inherited the majority of the BC characteristic peaks such as the O-H stretching peak at 3341 cm^− 1^, C-H stretching at 2891 cm^− 1^, C-O at C3; C-C stretching;, and C-O-H out of plan bending at 665 cm^− 1^. The intensity of the peak of C-O at C6 at 1054.

cm^− 1^ has been probably reduced for overlapping of the loaded materials and the change in the overall chemical composition of the nanofibrous matrix. Moreover, the composite sample showed fewer peaks distinguishing the MOF sample, where the most representative was the peak at 1366 cm^− 1^ [27].

In the Gr/Cu-Mg MOF spectrum, the occurrence of a peak at 1366 cm⁻¹ was assigned to symmetric stretching vibrations of the coordinated carboxylate groups (–COO⁻) of the BTC ligand, indicating successful coordination with Cu²⁺ and Mg²⁺ ions [39]. Besides, a band at 1620 cm⁻¹ attributed to C = C skeleton vibrations, and weak bands at 1220–1250 cm⁻¹ caused by C–O stretching of epoxide or alkoxy groups, revealed the presence of graphene oxide [40]. The absence of characteristic peaks of GO could be ascribed to the exfoliation and reduction of GO in ethanol by sonication during the synthesis procedure [41].

The X-ray diffraction patterns were used to recognize the crystalline structure of the BC, Gr/Cu-Mg MOF, and BC/Gr/Cu-Mg MOF samples as shown in Figure 2B. The BC sample exhibited two major diffraction peaks at 2θ ≈ 14.7° and 22.6° which corresponds to the (1̅10) and (200) planes of Cellulose I. These reflections support the considerably ordered and semi-crystalline framework of BC network corroborating the observations made on native BC fibers [42].

The XRD pattern for the Gr/Cu-Mg MOF showed a number of weak but resolvable diffraction peaks, suggesting the development of a semi-crystalline metal-organic framework. Somewhat weak peak intensity is usually attributed to partial crystallinity, which is a characteristic of bimetallic MOFs prepared under mild solvothermal conditions or containing GO. The presence of graphene tends to be problematic because it drastically disrupts the long-range order of the MOF lattice due to its high surface area and heterogenous nucleation tendency [43]. The XRD peaks of Cu-BTC (HKUST-1) were indexed using the reference card ICDD PDF# 00-064-1486., Unfortunately, there isn’t a universally recognized standard ICDD/JCPDS reference card number published for Mg-BTC (magnesium benzene-1,3,5-tricarboxylate MOF) in the common diffraction databases (like ICDD PDF) that’s widely cited in the literature, unlike Cu-BTC. the Mg-BTC structure was confirmed by comparison with literature-reported XRD patterns of Mg-BTC coordination polymers, due to the absence of an assigned PDF/JCPDS card [44].

For the case of BC/Gr/Cu-Mg MOF composite, the diffraction pattern was primarily dominated by the characteristic peaks of bacterial cellulose. The peaks at 14.7° and 22.6° were still sharp suggesting that the BC matrix’s crystalline structure was preserved after MOF deposition. Nevertheless, the lack or suppression of characteristic Gr/Cu-Mg MOF peaks in the composite is likely due to the lower loading or dispersal of MOF particles in the cellulose network. Such phenomena have also been discussed where in situ MOF growth on biopolymer substrates results in diminished peak intensity due to confinement and limited crystal growth [45]. As a whole, XRD analysis corroborates the synthesis of semi-crystalline Gr/Cu-Mg MOF and its incorporation in BC matrix, as it did not alter the structural crystallinity of BC.

**Fig. 3:**
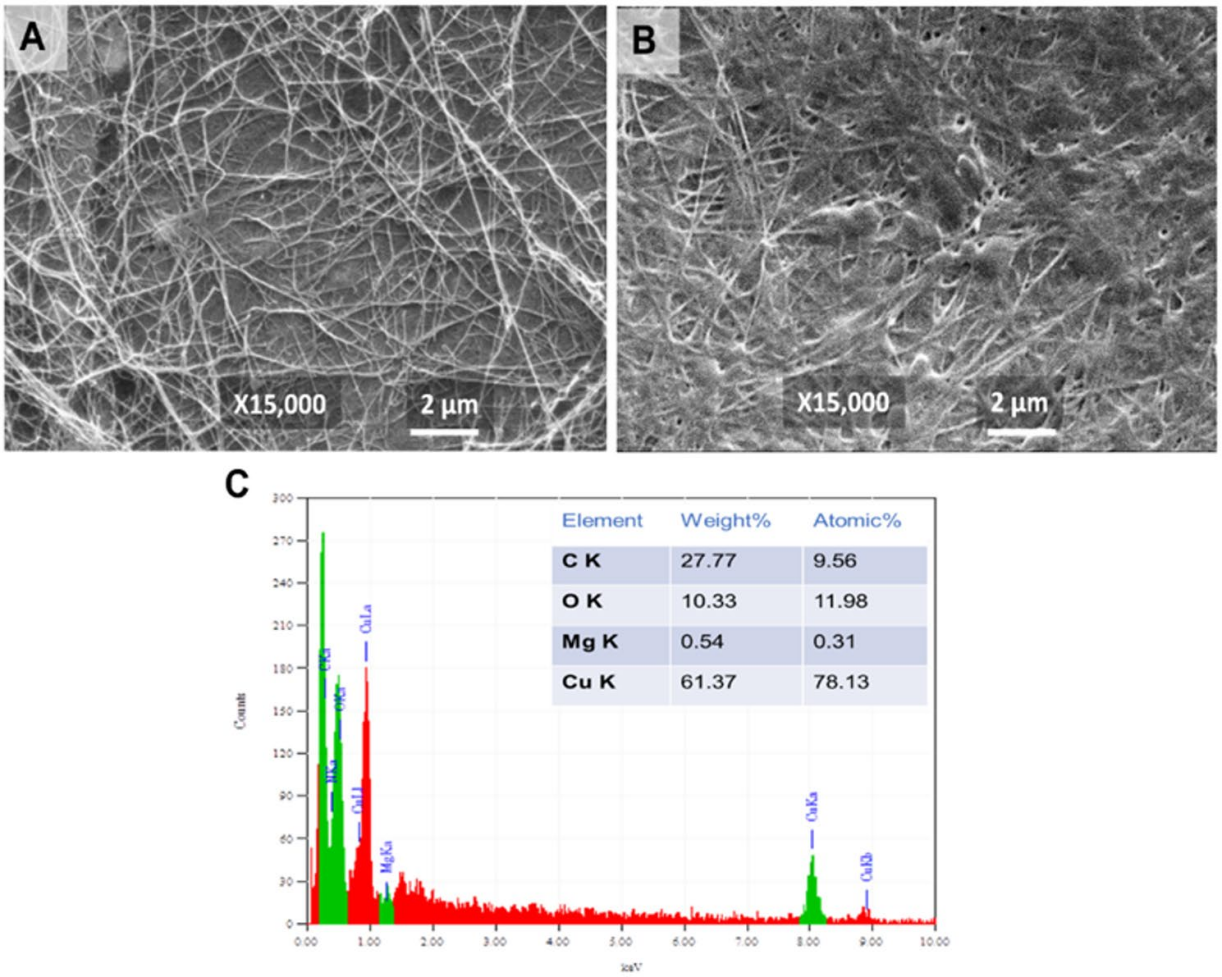
SEM investigation of the Gr/Cu-Mg MOF (**A**), and BC/Gr/Cu-Mg MOF (**B**); Area analysis by SEM-EDX for the BC/Gr/Cu-Mg MOF sample

Regarding the SEM, the ordinary 3D nanofibrillar web of the BC, where the nanopores of the nanostructure were evident (Fig. 3A). Whilst, the BC/Gr/Cu-Mg MOF sample showed up with depleted porosity. We presume that the Gr/Cu-Mg MOF has evolved around the BC nanofibers, causing either reduction of some BC pores size or totally block others (Fig. 3B). The EDX measurement revealed in Figure 3C confirmed the prominent subsistence of magnesium and copper elements in the BC/Gr/Cu-Mg MOF sample.

**Fig. 4:**
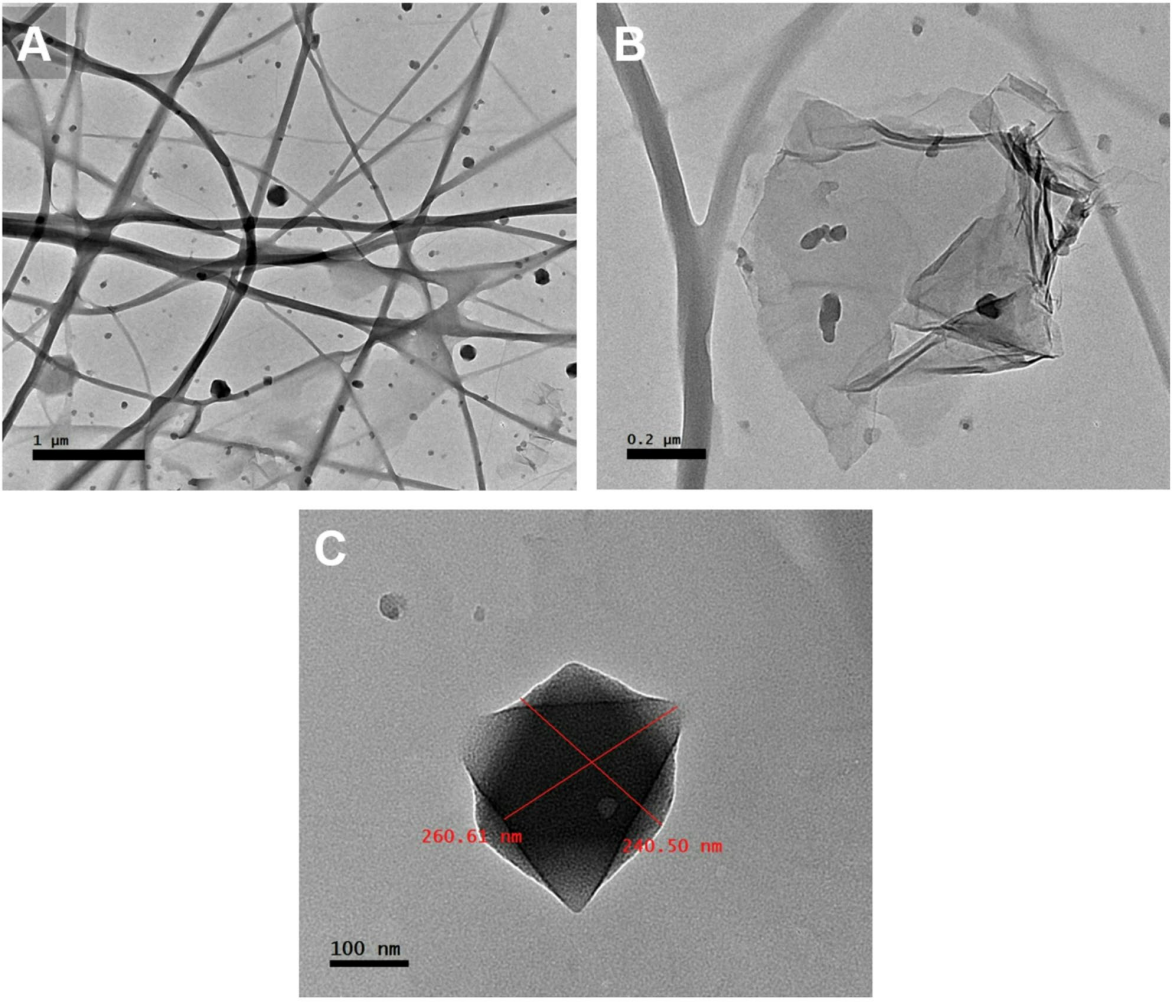
TEM determination of the BC/Gr/Cu-Mg MOF: graphene sheets/MOF immersed in BC nanofibers (**A**); graphene sheet elaboration (**B**); Cu-Mg MOF morphology and sizing (**C**)

Figure 4A elucidates the TEM image of the BC/Gr/Cu-Mg MOF nanocomposite disclosing the formation of the graphene nanosheets spangled with the Cu-Mg MOF nanoparticles. More obvious imaging for the graphene sheets is revealed in Figure 4B.

The TEM micrograph (Fig. 4C) reveals a well-defined Cu-Mg MOF particle with lateral dimensions of approximately 260 × 240 nm, exhibiting a diamond-like morphology. Such nanoscale structuring is consistent with the particle sizes reported for high-performance bimetallic MOFs synthesized under mild solvothermal conditions. The relatively uniform shape and compact structure suggest controlled nucleation and growth, which are critical for achieving reproducible physicochemical properties.

Recent studies support these observations. For instance, Ling et al. synthesized MgCu-MOF-74 *via* a one-pot method and reported particle sizes in the range of 200–300 nm, attributing the morphology to synergistic metal coordination and solvent-mediated crystallization [18]. Similarly, Kadi and his coworkers observed spherical and porous Cu-Mg MOF particles with average diameters below 100 nm, noting that magnesium incorporation modulates crystal growth and enhances surface area [43].

The absence of lattice fringes or high-contrast crystalline domains in the image may indicate partial crystallinity, a common feature in bimetallic MOFs, especially when incorporating graphene or other high-surface-area additives. This aligns with findings from Zhou et al., who reported diminished XRD peak intensity and disrupted long-range order in MOFs grown in situ on bacterial cellulose substrates due to confinement effects and heterogeneous nucleation [45].

**Fig. 5:**
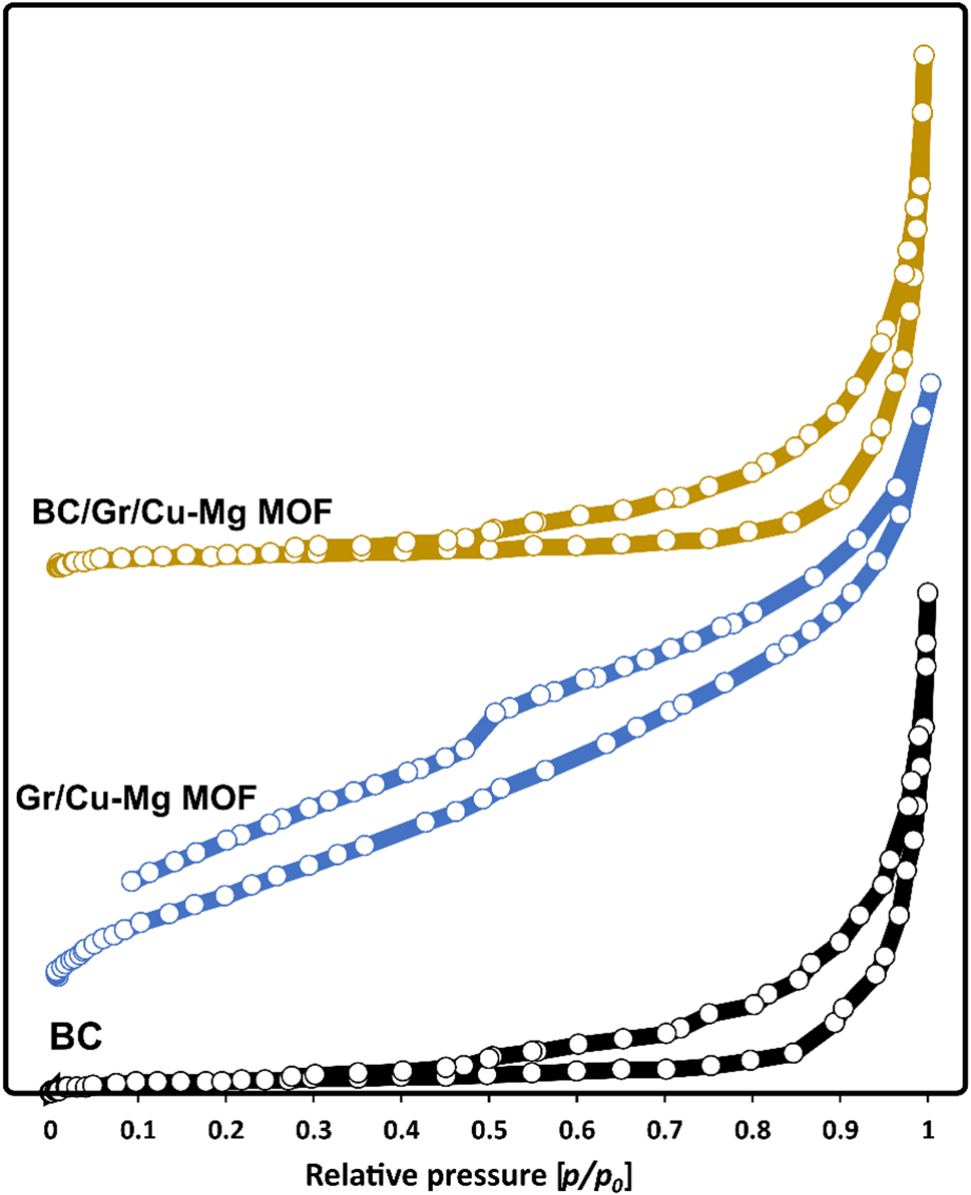
N_2_ adsorption/desorption isotherms of the BC, Gr/Cu-Mg MOF, and BC/Gr/Cu-Mg MOF

Brunauer–Emmett–Teller (BET) analysis was used to determine the specific surface area, pore volume, and average pore diameter of the BC, Gr/Cu-Mg MOF, and BC/Gr/Cu-Mg MOF samples. As depicted in Figure 5 and summarized in Table 3, pure BC possessed a surface area of 50.03 m²/g, a total pore volume of 0.19 cm³/g, and an average pore diameter of 22.16 nm, reflecting its inherent porous and nanofibrillar nature. The Gr/Cu-Mg MOF displayed a higher surface area of 75.23 m²/g owing to the micro- and mesoporous nature developed through the coordination of the metal ions with the BTC ligand. The Cu-Mg MOF, however, displayed a significantly lower total pore volume of 0.07 cm³/g and a smaller average pore diameter of 3.77 nm, indicating the formation of smaller and denser pores in the MOF structure. Once the nanocomposite material was established, the BC/Gr/Cu-Mg MOF showed a surface area of 61.7 m²/g, pore volume 0.11 cm³/g, and average pore diameter 15.8 nm.

These intermediate values demonstrate that MOF particles were successfully integrated into the BC network, filling partially its wider pores and reducing the average pore diameter while enlarging the specific surface area compared to BC alone. The BET results assured the successful growth o of Gr/MOF onto the BC matrix and demonstrated that the composite possesses a balance between available surface area and pore size, ideal for the efficient adsorption of metal ions from aqueous solution. These findings agree with the literature, where enhanced sorption properties have been observed by compositing MOFs with biopolymeric supports [35, 46, 47].

Regarding the tensile strength, the synthesized BC/Gr/Cu-Mg MOF nanocomposite showed, along with ductility and Young’s modulus, highly comparable with the corresponding values recorded by the pristine BC films (Table 4). We believe that the pinpointed mechanical features represent another evidence that the in situ strategy which employed for developing the BC/Gr/Cu-Mg MOF nanocomposite had mild or no effect on the BC moiety either on the chemical or crystalline levels.

**Table 3:** Surface area features of the BC, Gr/Cu-Mg MOF, and BC/Gr/Cu-Mg MOF samples

	Multipoint BET (m^2^/g)	BJH Total pore volume (cc/g)	Average pore diameter (nm)
BC	50.03	0.19	22.16
Gr/Cu-Mg MOF	75.23	0.07	3.77
BC/Gr/Cu-Mg MOF	61.7	0.11	15.8

**Table 4:** Mechanical properties of BC and BC/Gr/Cu-Mg MOF nanocomposite

Code	Tensile strength (MPa)	Elongation at break (%)	Young’s modulus (MPa)
BC	61 ± 4	3.5 ± 1.7	16 ± 3
BC/Gr/Cu-Mg MOF	62.8 ± 4.12	3.3 ± 2.1	15.1 ± 2.45

### Batch investigations

**Fig. 6:**
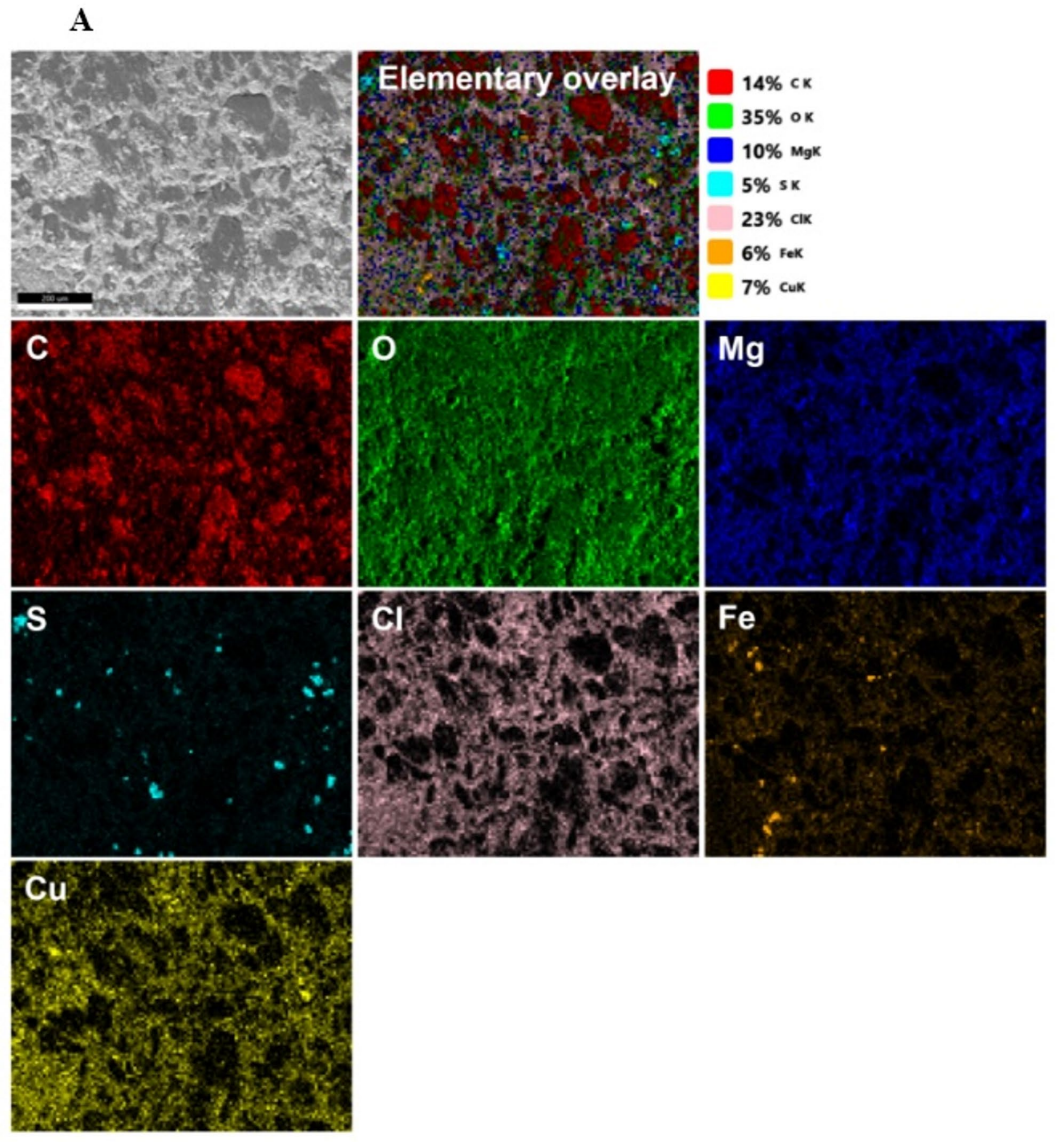

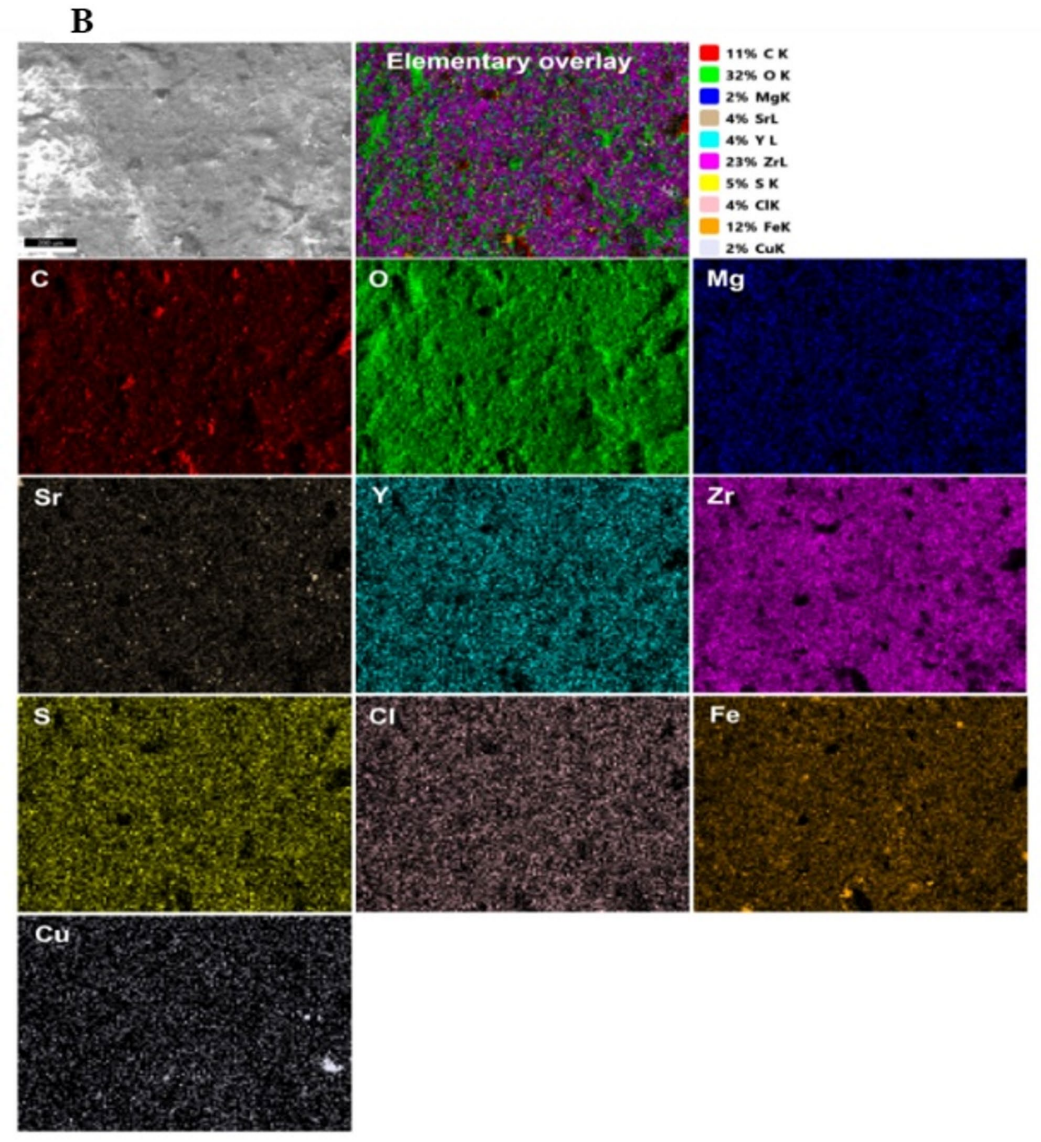
**A**,** B** represent the TEM-EDS analyses of the BC/Gr/Cu-Mg MOF before and after treatment of aqueous solution includes zirconium, yttrium, and strontium ions, respectively

Preliminary batch experiments were conducted to evaluate key factors influencing adsorption performance, including equilibrium time, pH, volume-to-mass (V/m) ratio, initial metal ion concentrations, and temperature. Figures 6A, B present EDS imaging of the BC/Gr/Cu-Mg MOF composite before and after treatment with the multicomponent ion system, respectively. Additionally, Figure S1 displays the corresponding EDX elemental analyses of the tested specimens.

#### Effect of aqueous solution pH

**Fig. 7:**
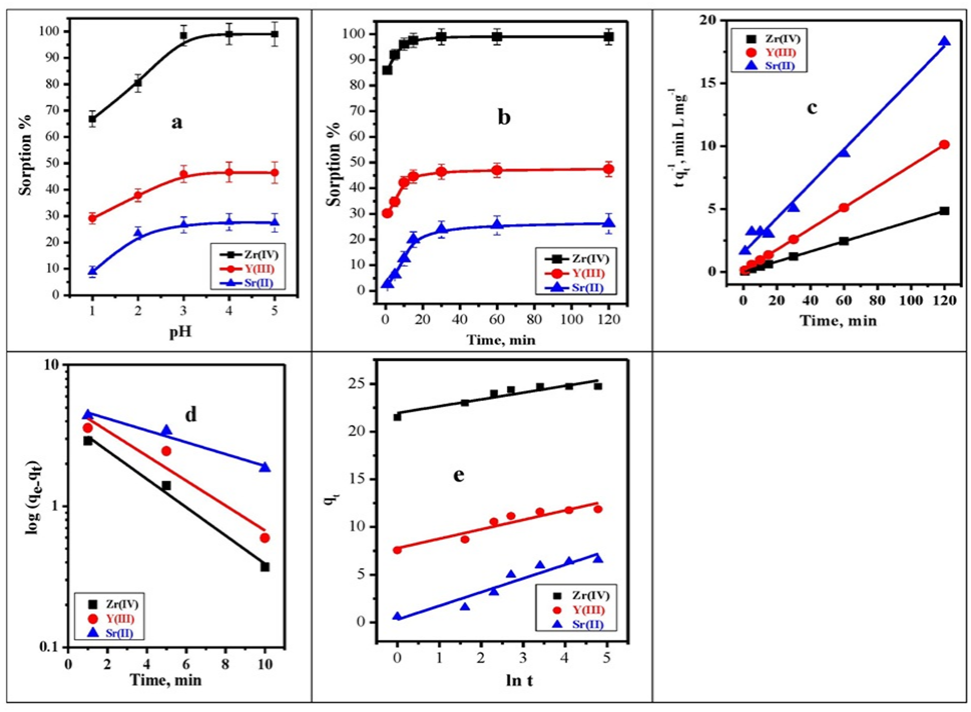
**a** Effect of the initial solution pH on the sorption percent of Zr^4+^, Y^3+^ and Sr^2+^ (t = 60 min, [M] = 100 mg L^− 1^, Dose = 0.02 g, V = 5 mL, T = 25 °C) from multicomponent system, **b** Effect of shaking time on the sorption percent of Zr^4+^, Y^3+^ and Sr^2+^, **c** Pseudo-second-order kinetic model, **d** Pseudo-first-order kinetic model, **e** Elovich Kinetic (pH = 3, [M] = 100 mg L^− 1^, Dose = 0.02 g, V = 5 mL, T = 25 °C) from multi-component system

The solution pH plays a critical role in controlling the adsorption efficiency of Zr(IV), Y(III), and Sr(II) ions, as it directly affects both the surface charge of the adsorbent and the protonation/deprotonation state of its functional groups. At low pH values (pH 1.0 − 2.0), the adsorbent surface is highly protonated, resulting in a positively charged surface that generates electrostatic repulsion toward the cationic metal ions, limiting adsorption. As the pH increases to 3.0, deprotonation of surface functional groups such as –OH, –COOH, and –NH₂ occurs, leading to a more negatively charged surface that favors electrostatic attraction, complexation, and chelation with Zr^4+^, Y^3+^, and Sr^2+^ ions. This behavior is consistent with the experimental data (Fig. 7a), which show that adsorption percentages increase sharply from pH 1.0 to 3.0 : Zr(IV) from 66.87% to 98.43%, Y(III) from 29.12% to 45.99%, and Sr(II) from 8.9% to 26.67%. Beyond pH 3, only slight improvements were observed (up to pH 5), indicating that most active sites were already available for metal ion binding. The enhanced adsorption at optimal pH 3.0 can thus be attributed to a combination of electrostatic interactions, coordination, and chelation mechanisms between the metal ions and the deprotonated functional groups on the adsorbent surface. Accordingly, pH 3.0 was selected as the optimal condition for subsequent adsorption experiments involving Zr^4+^, Y^3+^, and Sr^2+^ ions.

#### Effect of shaking time

Figure 7b displays the impact of shaking time over 120.0 min on Zr^4+^, Y^3+^, and Sr^2+^ ions adsorption efficiency on BC/Gr/Cu-Mg MOF adsorbent from a multicomponent system. The percentages of Zr^4+^, Y^3+^, and Sr^2+^ ions that were adsorbed increased over time until they reached adsorption equilibrium in 15.0 min. After that, the adsorption efficiency stayed the same for 120 min. This is because there will be a substantial number of binding sites available for achieving contact with the Zr^4+^, Y^3+^, and Sr^2+^ ions at the initial stage [48]. When saturation is reached, the binding sites will be full and won’t be able to interact with any more Zr^4+^, Y^3+^, or Sr^2+^ ions. The adsorption kinetics of Zr(IV), Y(III), and Sr(II) ions were investigated using pseudo-first-order (PFO), pseudo-second-order (PSO), and Elovich models (Table 5; Fig. 7c–e). Although the PFO model showed moderate correlation coefficients, the calculated equilibrium adsorption capacities were much lower than the experimental values, indicating its inadequacy in describing the kinetic behavior. In contrast, the PSO model provided an excellent fit with high correlation coefficients (R^2^ ≥ 0.989) and, more importantly, a close agreement between calculated and experimental adsorption capacities. This consistency confirms that the adsorption kinetics are best described by the PSO model and are mainly controlled by chemisorption involving interactions between metal ions and surface functional groups. The Elovich model exhibited lower R^2^ values, suggesting a limited contribution related to surface heterogeneity. Therefore, the adsorption mechanism was evaluated based on both kinetic parameters and capacity comparison rather than R^2^ values alone.

**Table 5:** Kinetic model parameters for Zr^4+^, Y^3+^, and Sr^2+^ adsorption from a multicomponent system ([M] = 100 mg L^− 1^, pH = 3, Dose = 20 mg, V = 5.0 mL, T = 25 °C)

Kinetic factor	Parameters	Metal ions		
		Zr(IV)	Y(III)	Sr(II)
Pseudo-First order	q_e_ (mg/g)	3.917	5.129	5.07
	K_1_ (g/mg.min)	0.231	0.203	0.097
	R^2^	0.979	0.869	0.938
Pseudo-Second order	q_e_ (mg/g)	24.81	11.98	7.30
	K_2_ (g/mg.min)	0.170	0.070	0.0122
	R^2^	0.999	0.999	0.989
Elovich model	${\upalpha}$	1.41	1.01	0.696
	${\upbeta}$	1.988 × 10^13^	2560.04	1.762
	R^2^	0.839	0.867	0.894

#### Effect of initial metal ion concentration

**Fig. 8:**
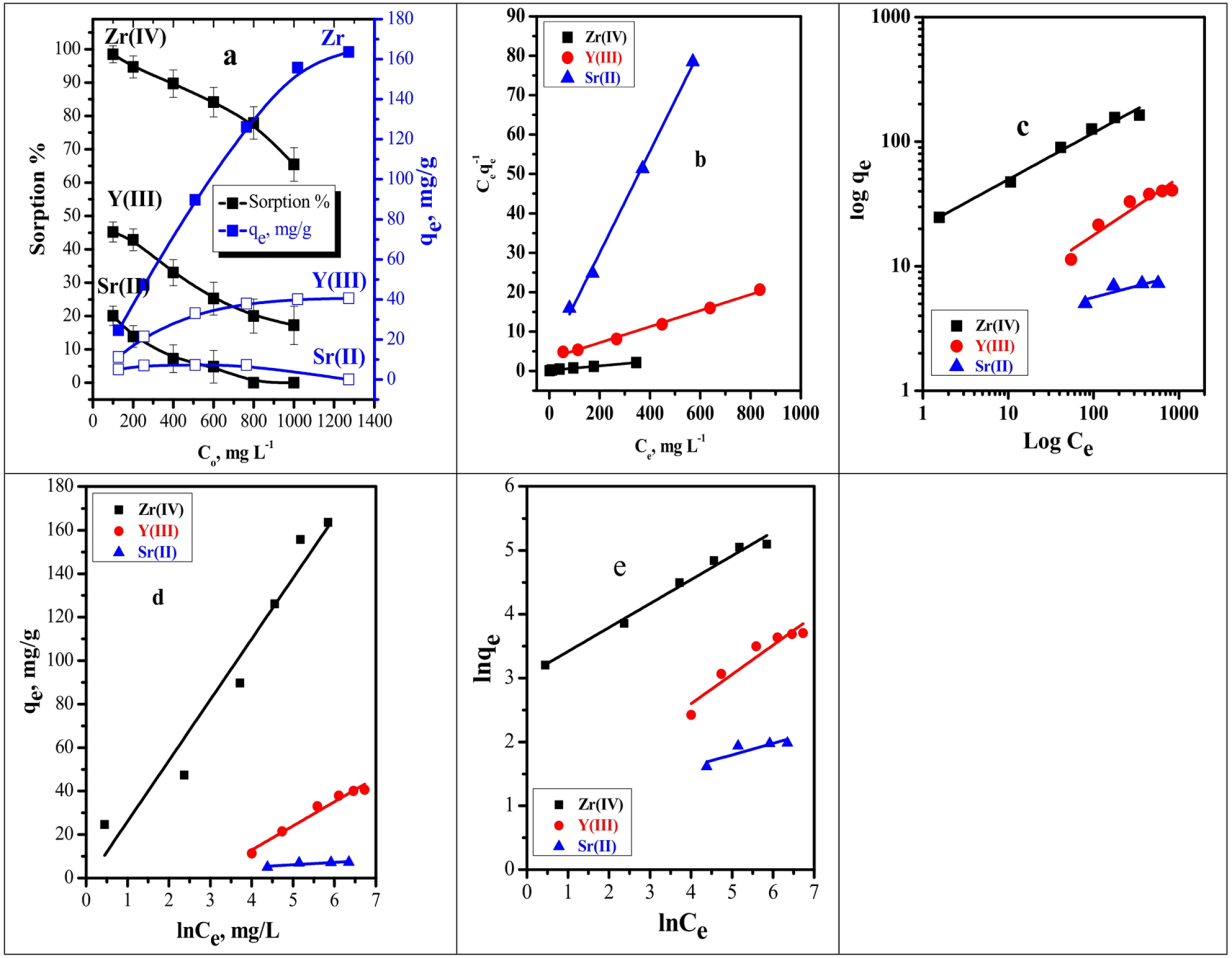
**a** Effect of initial metal ion concentration for the sorption percent and sorption capacities; **b** Linear Langmuir isotherm plots; **c** Linear Freundlich isotherm plots; **d** Linear Temkin isotherm plots; **e** Linear Halsey isotherm plots for the sorption percent of Zr^4+^, Y^3+^ and Sr^2+^( t = 15.0 min, pH = 3, Dose = 0.02 g, V = 5 mL, T = 25 °C from multicomponent system

The influence of the initial concentration of Zr^4+^, Y^3+^, and Sr^2+^ ions on the adsorption performance of the BC/Gr/Cu–Mg MOF composite was investigated within the concentration range of 100–1000 mg/L. As illustrated in Figure 8a, an inverse relationship between metal ion concentration and sorption efficiency (S%) was observed for all studied ions, whereas the sorption capacity increased progressively with increasing concentration. For Zr(IV), the sorption efficiency decreased from 98.44% at 100 mg/L to 65.43% at 1000 mg/L. In contrast, the sorption capacity increased substantially from 24.61 to 163.58 mg/g (Fig. 8a), indicating enhanced metal uptake at higher concentrations. A similar trend was recorded for Y(III), where S% declined from 45.21% to 17.24% with increasing concentration, while sorption capacity increased from 11.30 to 40.60 mg/g. These results confirm that although the percentage removal decreases at higher concentrations, the actual amount of metal ions adsorbed per unit mass of the sorbent increases. This decline in efficiency can be attributed to the saturation of available active binding sites on the adsorbent surface. At lower concentrations, the number of metal ions is relatively small, allowing sufficient interaction with the abundant functional groups on the composite. However, as the concentration increases, the competition among ions intensifies, surpassing the number of accessible adsorption sites, thereby reducing overall uptake efficiency, despite the higher driving force for mass transfer that promotes an increase in sorption capacity. In the case of Sr(II), a sharp decline in adsorption performance was observed, with complete inhibition occurring at concentrations ≥ 800 mg/L, reflecting its weaker interaction with the active sites of the composite compared to Zr(IV) and Y(III). Overall, these findings highlight that sorption capacity provides a more reliable measure for comparing adsorption performance at elevated metal ion concentrations than removal percentage, as it more accurately reflects the true uptake behavior of the adsorbent under equilibrium conditions.

To further interpret the adsorption behavior, the equilibrium data were analyzed using Langmuir, Freundlich, Temkin, and Halsey isotherm models (Fig. 8b–e). The correlation coefficients (R^2^) and parameters of the applied isotherm models are summarized in Table 6. The Langmuir model exhibited the best agreement with the experimental data, as evidenced by high correlation coefficients (R^2^ ≥ 0.992) for all investigated metal ions, indicating monolayer adsorption on a relatively homogeneous surface. The Langmuir maximum adsorption capacities (Q₀) were determined to be 175.44 mg/g for Zr(IV), 49.02 mg/g for Y(III), and 7.75 mg/g for Sr(II), which are consistent with the experimentally obtained adsorption capacities. The Langmuir affinity constant (b) reflects strong adsorbate–adsorbent interactions, while the dimensionless separation factor (R_L_) values between 0 and 1 confirm the favorability of the adsorption process. The Freundlich isotherm parameters showed adsorption intensity values (*n* > 1), suggesting favorable adsorption on heterogeneous surfaces. However, the comparatively lower correlation coefficients indicate that surface heterogeneity contributes less significantly than monolayer adsorption. The Temkin and Halsey models further support the presence of adsorbate–adsorbent interactions and multilayer adsorption behavior, particularly for Zr(IV) and Y(III). These findings confirm the composite’s high affinity and selectivity toward zirconium ions, followed by yttrium and strontium, under optimized conditions [49, 50].

**Table 6:** Langmuir and Freundlich parameters for the adsorption of Zr^4+^, Y^3+^ and Sr^2+^ ( t = 15.0 min, pH = 3, Dose = 0.02 g, V = 5 mL, T = 25 °C from multicomponent system

Isotherm	Parameters	Metal ions		
		Zr (IV)	Y(III)	Sr(II)
Langmuir	Qo (mg/g)	175.44	49.02	7.75
	b (ml/mg)	0.037	0.007	0.032
	R_L_	0.213	0.588	0,238
	R^2^	0.992	0.994	0.997
Freundlich	K_f_ (mg/g)	20.96	2.14	2.44
	n	2.67	2.18	5.54
	R^2^	0.983	0.899	0.659
Temkin	*K*_*T*_	0.932	0.058	1.75
	*B*	27.99	11.106	1.101
	R^2^	0.938	0.968	0.684
Halsey	n_H_	-2.67	-2.17	-5.54
	K_H_	0.884	0.545	0.817
	R^2^	0.982	0.899	0.66
q_exp_, mg/ g		178.05	50.67	7.275

#### Effect of adsorbent dosage

**Fig. 9:**
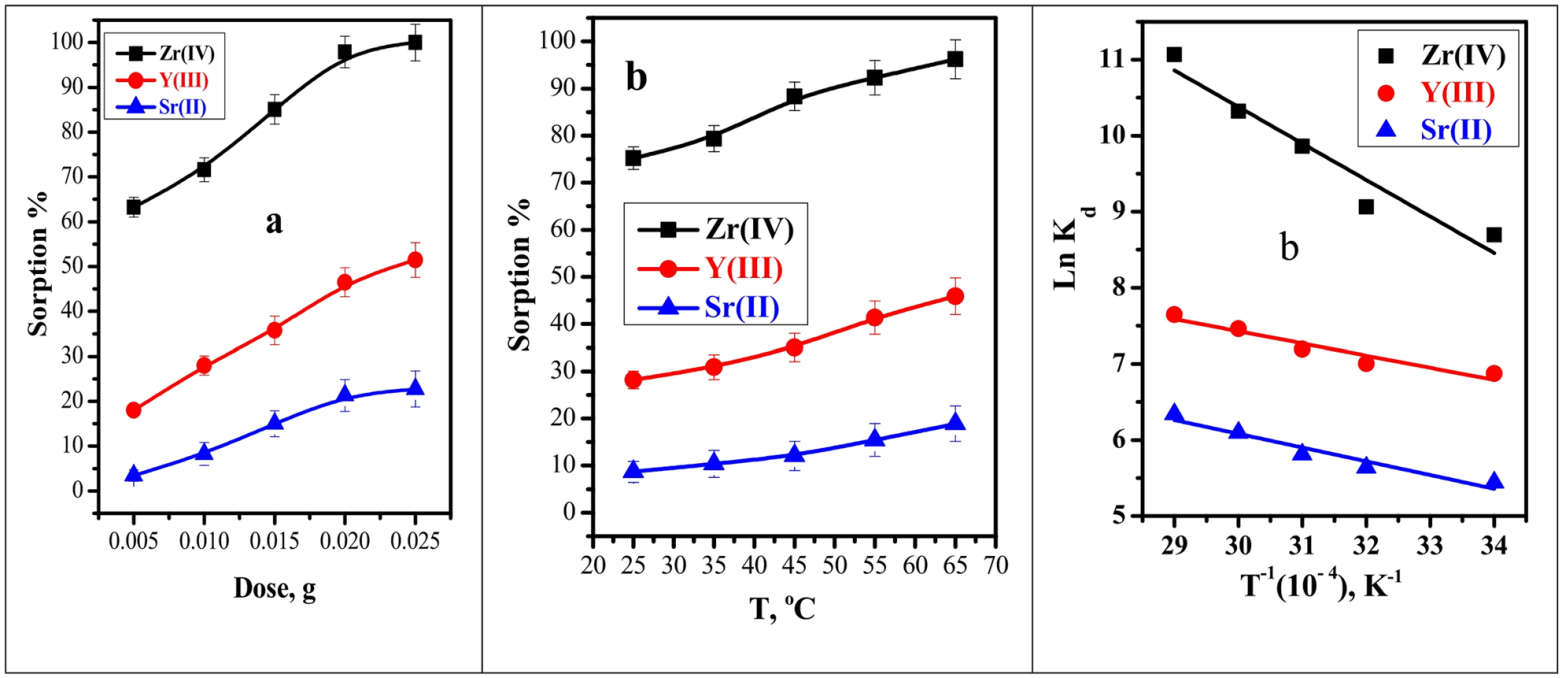
**a** Effects of adsorbent dosage on the sorption percent of Zr^4+^, Y^3+^ and Sr^2+^ ( t = 15.0 min, [M] = 100 mg L^− 1^, V = 5.0 mL, pH = 3.0, T = 25 °C)) from multicomponent system, **b** Effect of temperature on the sorption percent and **c** thermodynamic parameters of Zr^4+^, Y^3+^ and Sr^2+^ ( t = 15.0 min, [M] = 100 mg L^− 1^, V = 5.0 mL, pH = 3.0, Dose = 0.01 g)) from multicomponent system

Figure 9a depicts the impact of BC/Gr/Cu-Mg MOF adsorbent in the range of 0.005–0.025 g on the sorption efficiency of Zr(IV), Y(III), and Sr(II). As the adsorbent dosage increased from 0.005 to 0.025 g, the experimental results showed that the sorption percent increased from about 63.22 to 100% for Zr (IV), from about 18.01 to 51.46% for Y (III), and from 3.45% to 22.76% for Sr (II). This increase can be explained by the assumption that more active sites are available for the sorption of Zr(IV), Y(III), and Sr(II) ions when the adsorbent dosage increases [51].

#### Effect of solution temperature

Figure 9b reveals the effects of temperature on the BC/Gr/Cu-Mg MOF adsorption of Zr(IV), Y(III), and Sr(II) ions over a range of 25 to 65 °C. As the temperature increased, the results showed that the investigated metal ions sorption performance improved. This is because an increase in solution temperature facilitates the transfer of metal ion species from the bulk solution to the surface of the adsorbent. Thus, there is a greater chance of complexation between the metal ions and the functional groups on the adsorbent surface.

The thermodynamic behavior of Zr(IV), Y(III), and Sr(II) adsorption onto the BC/Gr/Cu–Mg MOF was evaluated using the linear plot of ln Kd versus 1/T (Fig. 9c), from which ΔG°, ΔH°, and ΔS° were calculated (Table 7). The negative values of ΔG° at 298–338 K indicate that the adsorption of Zr^4+^ (− 21.48 to − 29.73 kJ/mol), Y^3+^ (− 17.00 to − 21.05 kJ/mol), and Sr^2+^ (− 13.47 to − 17.30 kJ/mol) is spontaneous across the studied temperature range. The positive enthalpy changes, ΔH° (Zr^4+^ 39.97 kJ/mol, Y^3+^ 13.24 kJ/mol, Sr^2+^ 15.05 kJ/mol), confirm the endothermic nature of the adsorption process, indicating that higher temperatures favor metal uptake. This implies that the uptake of metal ions is favored by increasing temperature, as thermal energy helps overcome activation barriers and promotes the interaction between the ions and the functional groups of the adsorbent. Additionally, the positive entropy changes, ΔS° (Zr^4+^ 206.2 J/mol/K, Y^3+^ 101.46 J/mol/K, Sr^2+^ 95.71 J/mol/K), reflect increased randomness at the solid–solution interface during adsorption, suggesting enhanced affinity of the adsorbent toward the metal ions as the system becomes more disordered.

**Table 7:** Thermodynamic parameters for sorption of Zr^4+^, Y^3+^ and Sr^2+^ ions

T (K)	ΔG (kJ/mole)			ΔH(kJ/mole)			ΔS (J/mole/K)		
	Zr^4+^	Y^3+^	Sr^2+^	Zr^4+^	Y^3+^	Sr^2+^	Zr^4+^	Y^3+^	Sr^2+^
298	– 21.48	– 17.0	– 13.47	39.97	13.24	15.05	206.2	101.46	95.71
308	– 23.54	– 18.01	– 14.43						
318	– 25.60	– 19.02	– 15.39						
328	– 27.66	– 20.04	– 16.34						
338	– 29.73	– 21.05	– 17.30						

#### Comparison with other adsorbent material

The adsorption efficiency of BC/Gr/Cu-Mg MOF for Zr^4+^, Y^3+^, and Sr^2+^ ions in aqueous solutions was evaluated by comparing its capacity with various adsorbent materials reported in the literature (Table 8). The results indicate that BC/Gr/Cu-Mg MOF exhibits a significantly higher adsorption capacity for these metal ions compared to other materials. This suggests that BC/Gr/Cu-Mg MOF is a highly effective adsorbent for removing these metals from aqueous solutions.

**Table 8:** Adsorption capacities of different adsorbents of Zr^4+^, Y^3+^ and Sr^2+^

Adsorbate	Adsorbent	q_o_(mg/g)	Refs.
	polyacrylic-carboxymethyl cellulose-trioctyl amine/reduced grapheme oxide adsorbent (AA-CMC-TOA/rGO)	90.31	[52]
	Mono-phosphate functionalized silica gel (MPFSG)	12.3	[53]
	Bi-phosphate functionalized silica gel (BPFSG)	16.7	[53]
	Amine, amido pyridyl phosphate functionalized silica gel (AAPPFSG)	29.0	[53]
	Zeolite NaX	75.0	[54]
	BC/Gr/Cu-Mg MOF	175.44	This work
Y^3+^	polyacrylic-carboxymethyl cellulose-trioctyl amine/reduced grapheme oxide adsorbent (AA-CMC-TOA/rGO)	95.13	[52]
	Ion imprinted polymers Y(III)-IIPs	10.26	[55]
	Pectin-Chitosan	23.0	[56]
	Sulfadiazine Schiff base (SDSB)	80.01	[57]
	Chitin powder	67.24	[58]
	Chitin aerogel	92.33	[58]
	Durian rind biosorbent	35.0	[59]
	Magnetic Silica Hybrid Material with P507	7.34	[60]
	BC/Gr/Cu-Mg MOF	49.02	This work
Sr^2+^	Amberlite XAD–4 resin impregnated with Cyanex 572	6.1	[5]
	Crab carapace	3.92	[61]
	Dry cow dung powder	9.0	[62]
	Multiwall carbon nanotubes	1.62	[2]
	Oxidized multiwall carbon nanotubes	6.62	[2]
	Na_3_FePO_4_CO_3_	99.6	[63]
	BC/Gr/Cu-Mg MOF	7.75	This work

## Conclusions

The present study contributes to the development of innovative nanocomposites based on sustainable materials, aimed at advancing environmental remediation strategies for ecosystem restoration. A novel multicomponent system comprising bacterial cellulose, graphene, and a bimetallic Cu–Mg metal-organic framework (BC/Gr/Cu–Mg MOF) was synthesized and tailored for the adsorption of zirconium, yttrium, and strontium ions from aqueous media. The synthesis approach adhered to green chemistry principles, optimizing both time and cost efficiency without compromising the physicochemical integrity or homogeneity of the resulting nanocomposite. Adsorption behavior was rigorously evaluated, with the Langmuir isotherm model confirming the material’s high affinity and capacity for the targeted metal ions. Batch adsorption experiments revealed empirical capacities of 178.05 mg/g for Zr(IV), 50.67 mg/g for Y(III), and 7.28 mg/g for Sr(II). Adsorption kinetics followed the pseudo-second-order model, with calculated equilibrium capacities of 24.81, 11.98, and 7.30 mg/g for Zr(IV), Y(III), and Sr(II), indicating that chemisorption governs the adsorption process. Key operational parameters including solution pH, contact time, initial ion concentration, adsorbent dosage, and temperature were systematically investigated to elucidate their influence on sorption performance. Future research will focus on continuous batch experiments and scaling up the system to assess its viability for industrial applications.

## Supplementary Information

Below is the link to the electronic supplementary material.

Supplementary Material 1.

## Data Availability

All data generated or analyzed during this study are included in this published article and its supplementary information files.
