# Supplementary material for: Bacterial cellulose nanofibers decorated with graphene/Cu-Mg MOF for sorption of zirconium, yttrium, and strontium ions from multicomponent system
Source: BMC Chem. 2026 Apr 9;20(1):85. doi: 10.1186/s13065-026-01774-5 (PMC13085723; doi:10.1186/s13065-026-01774-5)
Supplement: Supplementary file 1 — Supplementary Material 1. [file 13065_2026_1774_MOESM1_ESM.docx]

**Supplementary Material**

**Bacterial cellulose nanofibers decorated with graphene/Cu-Mg MOF for sorption of** **zirconium, yttrium, and strontium ions from multicomponent system**

**E.M. Abu Elgoud* ^1^, Aya M. Matloob ^2^, Deyaa Abol-Fotouh ^3^, H.F. Aly ^1^,**

**& Ola E. A. Al-Hagar ^4^**

^1)^ Nuclear Fuel Chemistry Department, Hot Laboratories Center, Egyptian Atomic Energy Authority, 13759, Egypt.

^2)^ Refining department, Egyptian Petroleum Research Institute (EPRI), Naser City, 11727, Cairo, Egypt.

^3)^ Advanced Technology and New Materials Research Institute (ATNMRI), City of Scientific Research and Technological Applications (SRTA-City), New Borg El-Arab City, Alexandria 21934, Egypt.

^4)^ Plant Research Department, Nuclear Research Center, Egyptian Atomic Energy Authority, Cairo 13759, Egypt.

^*^Corresponding author, email: [elsayedmustafa36@yahoo.com](mailto:elsayedmustafa36@yahoo.com), **Tel. (+2) 01552734381**

**Figure S1:** A & B represent the EDX analysis of the BC/Gr/Cu-Mg MOF before and after
treatment of aqueous solution includes zirconium, yttrium, and strontium ions, respectively.


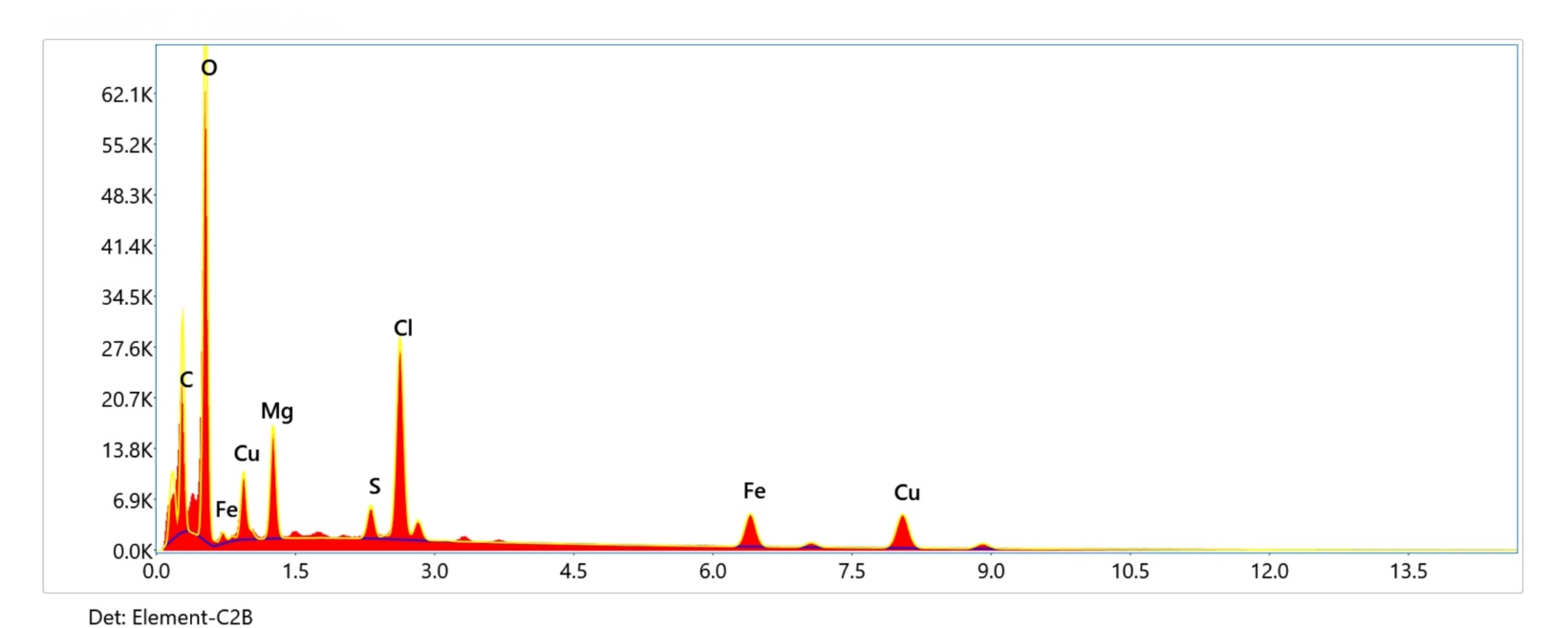

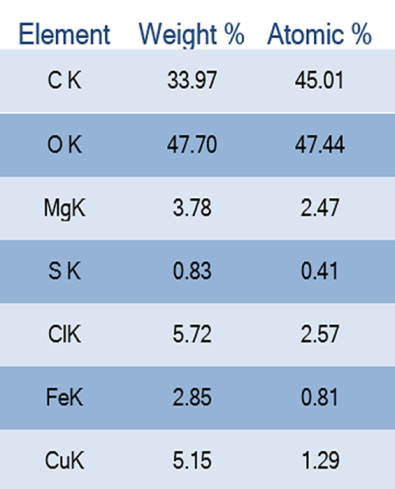


**A**


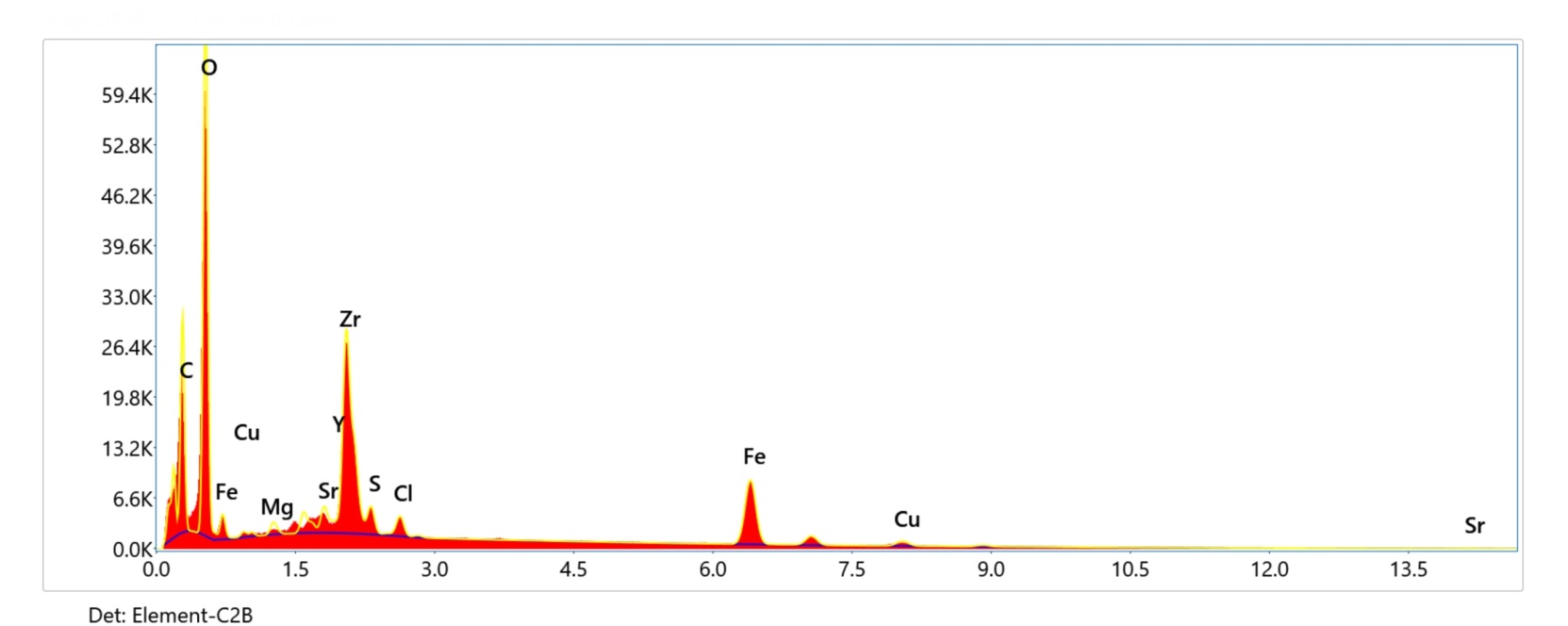

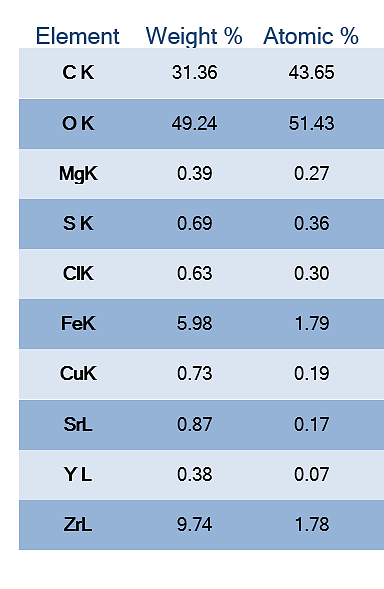


**B**
